# Mucosal microbiome of surgically treated terminal ileal Crohn’s disease

**DOI:** 10.3389/fcimb.2023.1324668

**Published:** 2024-01-12

**Authors:** Florian N. Loch, Carsten Kamphues, Peter Menzel, Rolf Schwarzer, Katharina Beyer, Christian Schineis

**Affiliations:** ^1^ Department of Surgery, Charité – Universitätsmedizin Berlin, Corporate Member of Freie Universität Berlin and Humboldt-Universität zu Berlin, Berlin, Germany; ^2^ Department of Surgery, Park-Klinik Weißensee, Berlin, Germany; ^3^ Labor Berlin – Charité Vivantes GmbH, Berlin, Germany

**Keywords:** Crohn’s disease, microbiome, surgery, ileocecal resection, mucosa

## Abstract

Crohn’s disease (CD) is associated with changes in the microbiome. The role of these changes and their precise association with disease course and activity remain ambiguous. In this prospective single-center study, the mucosal microbiome of surgical CD and non-CD patients was compared at the time of surgery. Microbial analyses were individually performed for ileal and colonic tissue samples obtained during surgery using 16S-rRNA-gene amplicon sequencing. Three groups out of the 46 included patients were formed: 1) a study group of CD of patients who received ileocecal resection due to CD involvement (CD study, n=10); 2) a control group of non-CD of patients who received intestinal resection due to indications other than CD (non-CD control, n=27); and 3) a second control group of CD who underwent resection of the intestine not affected by CD (CD non-affected control, n=9). Species richness and Shannon diversity were not different between all formed groups and regions analyzed (p>0.05). Several significant taxonomic differences were seen at the phylum-, order-, and genus-levels between the formed groups, such as a decrease of *Firmicutes* (phylum-level) and an increase of *Bacteroides* and *Escherichia/Shigella/Pseudescherichia* (genus-level) in CD study – colon vs. the non-CD control – colon (p ≤ 0.05). The CD non-affected control presented the largest amount of differentially abundant taxa in comparison to the other groups. These results underline that CD is accompanied by changes in affected and non-affected intestinal regions compared to non-CD controls. This study contributes the mucosal microbiome of a well-defined subset of surgical CD patients without confounding aspects of the fecal microbiome or regional microbial differences to the existing literature.

## 1 Introduction

In Crohn’s disease (CD), a fine interplay of genetic susceptibility and external promoting factors underlies the elicited immune responses that lead to intestinal inflammation (Sartor, 2006). A key factor for a healthy homeostasis of intestinal immune responses is an extensive and precise interaction between the present microbiome andthe mucosal immune system (Wu and Wu, 2012). In this context, recent studies have provided insights into the association between alterations in the intestinal microbiome and CD (Wright et al., 2015). To date, it remains ambiguous if these changes in the microbial composition are a causing factor, pathophysiologically promoting CD, or a correlating factor caused by disease onset and activity (Wright et al., 2015). However, changes in microbial composition have been shown to have the potential to diagnose new-onset CD (Gevers et al., 2014) and disease activity (Zhou et al., 2018) and to predict disease response to anti-inflammatory therapy (Rajca et al., 2014; Doherty et al., 2018; Zhou et al., 2018).

Despite great progress made in anti-inflammatory therapy in the last decades, up to 80% of CD patients will require at least one surgery during their lifetime (Cosnes et al., 2011). Conditions for the necessity of such surgical interventions are disease refractory to anti-inflammatory therapy, the development of penetrating disease, and fibrotic stenosis (Meima-van Praag et al., 2021). Identifying and comprehending changes in the microbial profile of CD patients that are associated with or even precede the course of the disease and the development of such conditions may enhance treatment decisions and the timing and planning of surgery.

Existing literature on the microbiome of CD is often limited by the investigation of the fecal rather than tissue samples (Wright et al., 2015). Because mucosal and fecal microbial compositions differ (Momozawa et al., 2011), a fecal analysis may confound microbial profiles that actually interact with the mucosal immune system and are directly associated with CD (Altomare et al., 2019). Additionally, there are regional differences in the intestinal microbial composition that cannot be differentiated in fecal samples (Martinez-Guryn et al., 2019). Furthermore, studies focusing on patients in the context of surgery for CD are limited (Dey et al., 2013; Laffin et al., 2018; Sokol et al., 2020; Zhuang et al., 2021).

Thus, the aim of this study is the individual analysis and comparison of the mucosal microbial composition of ileal and colonic tissue samples obtained at the time of surgery from a study group of patients receiving ileocecal resection for CD, a control group of non-CD patients that underwent terminal ileal and right-sided ileocolic resection, and a second control group of CD patients receiving resection of the non-affected terminal ileum during closure of a protective ileostomy.

## 2 Materials and methods

### 2.1 Patients

In this prospective, single-center study approved by the institutional ethics committee (#EA4/165/18), patients were included who received surgery at the Department of Surgery, Campus Benjamin Franklin, Charité—Universitätsmedizin Berlin, between March 2019 and August 2020. Three groups of patients were formed: 1) a study group of patients who received ileocecal resection for CD (CD study); 2) a control group of patients who underwent terminal ileal resection, ileocecal resection or right-sided ileocolic resection for indications other than CD and who were not affected by ulcerative colitis (e.g., malignant tumors, closure of protective ileostomy, non-CD control); and 3) a second control group of CD patients who received resection of the non-affected terminal ileum as part of the closure of a protective ileostomy (CD non-affected control). This way, all ileal samples obtained for this study originated from the terminal ileum, and all colonic samples were from the right-sided colon (cecum or ascending colon). Inclusion criteria were one of these indications, age ≥ 18 years, and histologically confirmed CD for the study and second control groups. Sample exclusion criterion were bacterial load below the detection threshold as measured by next-generation sequencing, as described below. All patients included in this study received single-shot perioperative antibiotic prophylaxis within 60 minutes prior to surgery. For solely ileal resections, cefuroxime was used, and a combination of cefuroxime and metronidazole was used for resections involving the colon. In cases of allergies, cefuroxime was replaced by clindamycin. After application of the exclusion criterion, 54 patients were excluded, and a total of 76 samples from 46 patients were included in this study. The CD study consisted of 10 patients, the non-CD control involved 27 patients, and the CD non-affected control had a total of nine patients. Written informed consent was obtained from all patients before inclusion in this study.

### 2.2 Tissue sampling, DNA extraction, and microbiome sequencing

Two separate full-thickness tissue samples, one from the proximal and one from the distal intestinal resection margin (e.g., one from the proximal ileum and one from the distal cecum in an ileocecal resection), were obtained from each patient directly after intraoperative removal of the surgical specimen. DNA extraction, microbiome sequencing, and sequence analysis were performed as previously described (Fluhr et al., 2022): Obtained tissue samples were individually homogenized in PowerBead Pro Tubes (Qiagen, PowerSoil Pro-Kit) in a MagNA Lyser (Roche), followed by DNA extraction and elution in 50 µL elution buffer (Qiagen, DNeasy PowerSoil Pro-Kit). In each run, negative buffer controls and positive community standards were analyzed in parallel. The V3-V4 region of the 16S-rRNA gene was amplified with the ultra-clean production multiplex polymerase chain reaction (PCR) master mix (Qiagen) from 2 µL of DNA using the forward primer CCTACGGGNGGCWGCAG and reverse primer GACTACHVGGGTATCTAATCC based on the Illumina 16S library preparation protocol (https://support.illumina.com/documents/documentation/chemistry_documentation/16s/16s-metagenomic-library-prep-guide-15044223-b.pdf). A total of 2.6 pg of DNA from Salinibacter ruber (DSM 13855) was added as an internal spike-in control. Sequencing was done on an Illumina MiSeq (v2 reagents) with 2 × 250 bp paired end reads.

### 2.3 Sequence analysis

The standard UPARSE 16S protocol was used after sequencing to merge paired reads by their 3′-ends, filter reads to a minimum length of 100 bp, cluster unique reads into operational taxonomic units (OTUs) based on a 97% identity threshold, and quantify OTU abundances by mapping reads to the OTU sequences (Edgar, 2013). OTUs with relative abundances of less than 0.01% were discarded. In order to assign OTUs to taxa, each OTU sequence was searched in the NCBI Targeted Loci 16S database (NCBI accession PRJNA33175) using NCBI BLAST (Camacho et al., 2009) with an identity cut-off of 97% and an E-value cut-off of 0.01. If no match was found for an OTU sequence in this way, it was additionally searched in the NCBI BLAST NT database using the same cut-offs, but excluding unclassified species or environmental sequences (based on the taxon names in the NCBI taxonomy). If no match was found for an OTU in both databases, it was marked as unclassified. OTUs with multiple best matches were assigned to the least common ancestor of all alignments with the same BLAST bit score. OTUs assigned to the *Salinibacter ruber* spike-in were discarded. Samples with a minimum of 100 reads assigned to bacterial OTUs and at least twice as many bacterial reads compared to the buffer-negative controls were considered positive and included in the analysis. The diversity and heterogeneity of a single microbiome sample can be measured by various diversity indices. Here, species richness (number of OTUs in each sample) and Shannon diversity were calculated as measures of α-diversity. The Shannon diversity index H is defined as H = − ∑_i_ p_i_ · ln p_i_, where p_i_ represents the proportion of OTU i relative to the total number of OTUs in a sample. Relative abundances for taxa were summarized at the ranks of genus, order, and phylum and calculated from classified OTUs for these ranks. The visualization of microbiome data was done using ggplot2 (Wickham, 2009). Principal Coordinate Analyses (PCoAs) were performed using the Bray-Curtis dissimilarity measure on OTU relative abundances, using the R packages vegan and ecodist.

### 2.4 Statistical analysis

Medians of species richness (OTUs) and Shannon diversity indices were calculated. Mean relative abundance (MRA) was calculated for taxa at the phylum-, order-, and genus-level of each individual group of patients and intestinal region (i.e., ileum and colon) with respective 95% confidence intervals (CIs). The Wilcoxon rank sum test was used to compare α-diversity and Shannon diversity indices between individual groups and for pairwise comparison of mean relative abundances (MRAs) of taxa. A p-value of ≤0.05 was considered statistically significant, with p-values of ≤0.10 taken as a trend toward significance.

## 3 Results

The composition of the formed groups of all patients included in this study, as well as the respective demographic and clinical characteristics, are summarized in **Table 1**. General taxonomic analysis of ileal vs. colonic samples revealed a significant difference in species richness along with a significant variation in the MRA of the three most abundant phyla (**Supplementary Figure 1**, p ≤ 0.05).

**Table 1:** Demographic and clinical characteristics of the study group of CD patients and the control groups.

	CD patients n=10	Non-CD patients n=27	CD patients with non-affected ileum n=9
**Age (median in years)**	39 (29-61)	69 (29-86)	35 (19-61)
**Female patients**	5 (50.0%)	11 (40.7%)	3 (33.3%)
**BMI**	21.4 (18.0-26.0)	23.5 (18.4-29.7)	24.8 (15.6-34.7)
**Ileal samples**	9	33	11
**Colonic samples**	10	13	–
**Time from the initial diagnosis of CD to surgery (median in years)**	12 (1-36)	–	10 (1-19)
Phenotype of CD			
Stricturing	6 (60.0%)	–	–
Penetrating	1 (10.0%)	–	–
Stricturing and penetrating	3 (30.0%)	–	–
**Medical therapy for CD at the time of surgery**			
None	2 (20.0%)	–	5 (55.6)
Mesalazine	2 (20.0%)	–	1 (11.1%)
Prednisone	2 (20.0%)	–	1 (11.1%)
Azathioprine and Biologics (infliximab, adalimumab)	1 (10.0%)	–	
Biologics (infliximab, adalimumab, vedolizumab)	3 (30.0%)	–	2 (22.2%)

CD, Crohn’s disease.

### 3.1 Taxonomy of CD study vs. non-CD control

**Figure 1:**
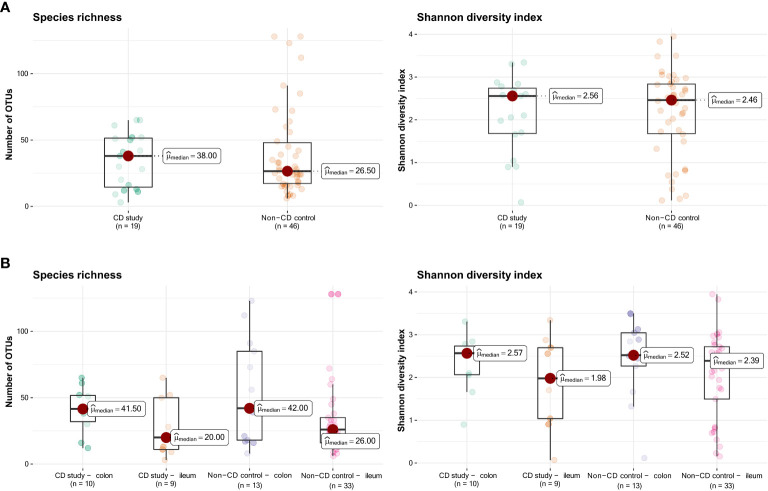
Species richness (left) and Shannon diversity index (right) of the CD study vs. the non-CD control. **(A)** For the entire group of the CD study vs. the non-CD control. **(B)** For ileal and colonic samples individually. OTUs: operational taxonomic units.

**Figure 1** presents the species richness and Shannon diversity index of the CD study vs. the non-CD control for ileal and colonic samples pooled together (**Figure 1A**) and individually (**Figure 1B**). No difference was seen in α-diversity for species richness and Shannon diversity index, overall (**Figure 1A**), and for ileal and colonic samples individually (**Figure 1B**, p>0.05).

**Figure 2:**
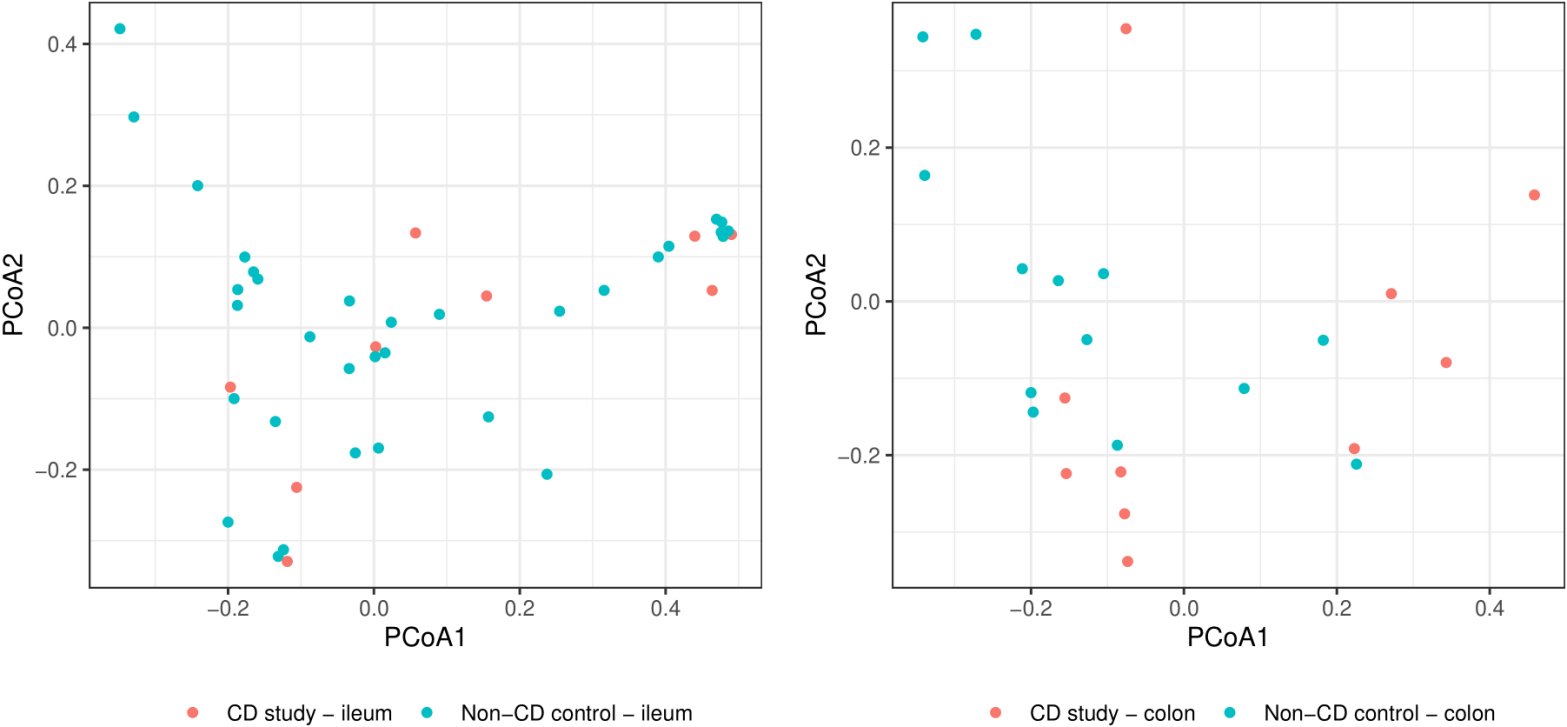
Principal coordinate analyses (PCoAs) for overall discrimination of β-diversity of the CD study vs. the non-CD control for ileal samples (left) and colonic samples (right) based on Bray-Curtis dissimilarity.

**Figure 3:**
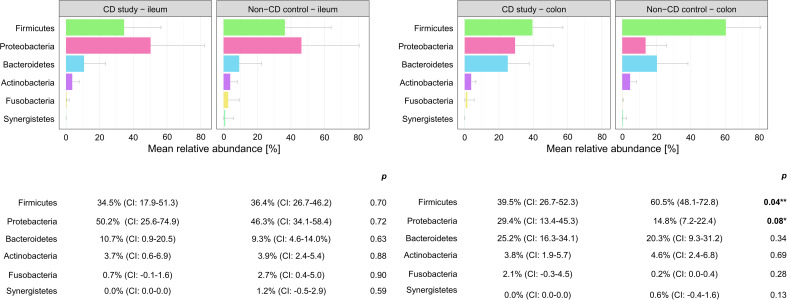
Discrimination of the microbial composition of Crohn’s disease patients from the CD study and the non-CD control at the phylum level for ileal and colonic samples. Mean relative abundances are presented with corresponding confidence intervals (CIs) and p-values comparing the differences between the two groups from the Wilcoxon rank-sum test (**p-values ≤ 0.05; *p-values ≤ 0.10). CI, confidence interval.

**Figure 4:**
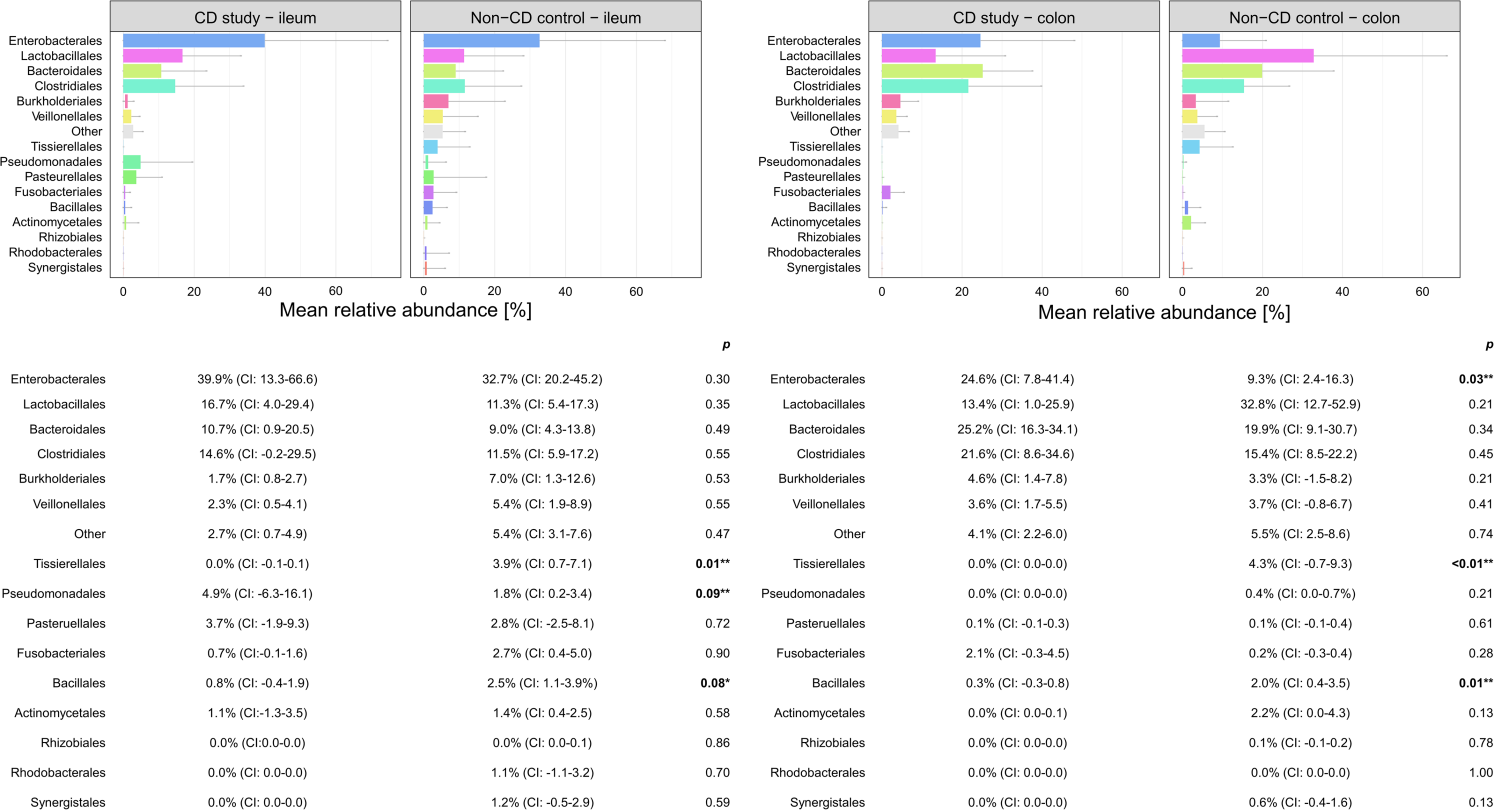
Discrimination of the microbial composition of Crohn’s disease (CD) patients from the CD study and the non-CD control at the order level for ileal and colonic samples. Mean relative abundances are presented with corresponding confidence intervals (CIs) and p-values comparing the differences between the two groups from the Wilcoxon rank-sum test (**p-values ≤ 0.05; *p-values ≤ 0.10). CI, confidence interval.

**Figure 5:**
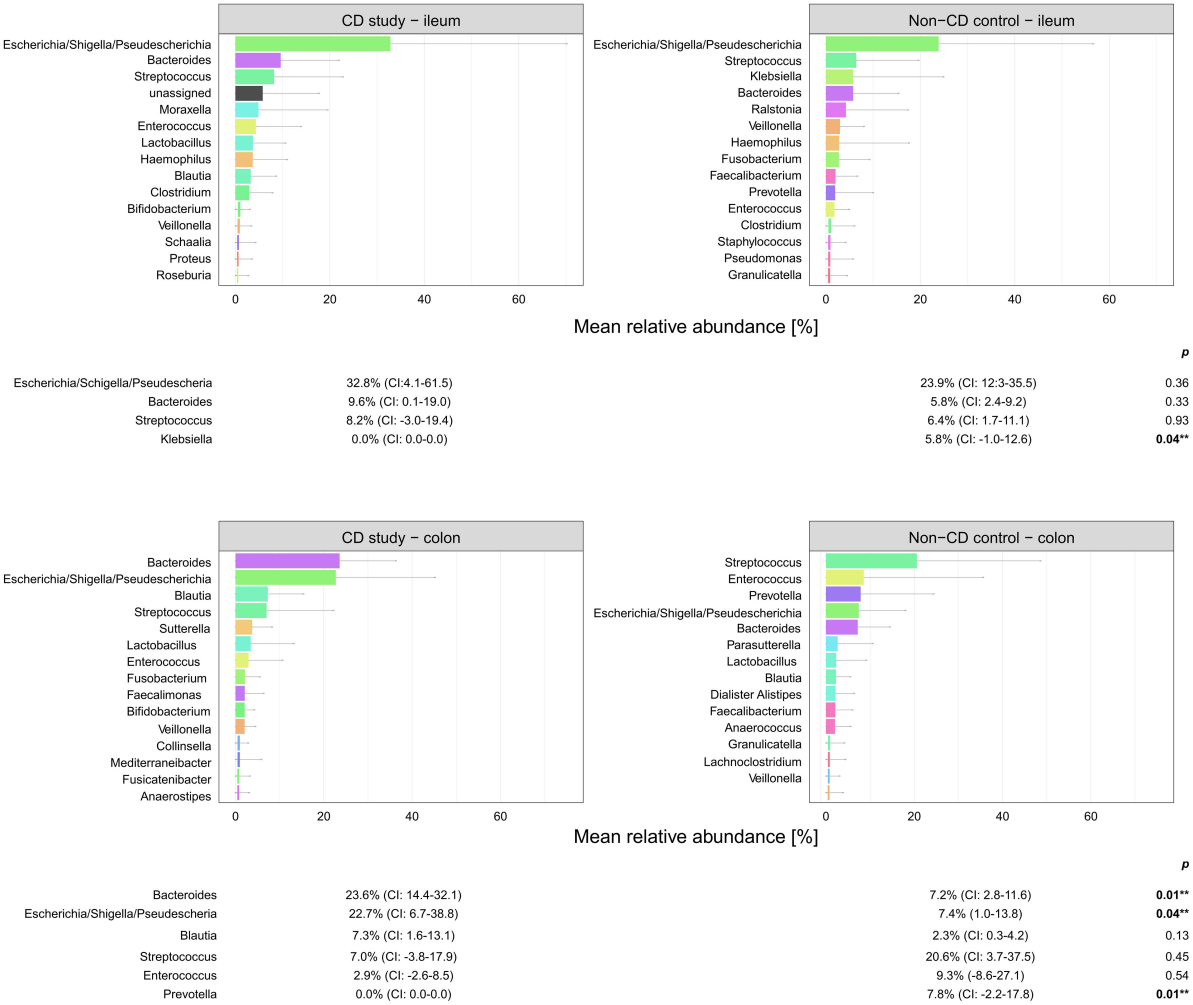
Microbial composition of Crohn’s disease (CD) patients from the CD study and the non-CD control at the genus level for ileal and colonic samples. For genera with mean relative abundances (MRAs) of ≥ 5.0% in at least one of the groups, MRAs are presented with corresponding confidence intervals (CIs) and p-values comparing the differences between the two groups from the Wilcoxon rank-sum test (**p-values ≤ 0.05). CI, confidence interval.

In order to further discriminate the taxonomic profiles, **Figure 2** presents PCoAs of β-diversity of the CD study vs. the non-CD control for ileal samples and colonic samples. MRAs of phylum-level taxa from the CD study and the non-CD control are shown and compared individually for ileal and colonic samples in **Figure 3**. Subsequently, MRAs at the order-level and the genus-level are presented in **Figures 4**, **5**, respectively.

In ileal samples, *Tissierellales* (order-level) and *Klebsiella* (genus-level) were only present in the non-CD control – ileum (0.0% (CI:-0.1-0.1) vs. 3.9% (CI: 0.7-7.1%) and 5.8% (CI: -1.0-12.6%), p<0.05). *Pseudomonadales* were more abundant and *Bacillales* less abundant in the CD study – ileum compared to the non-CD control – ileum with a trend toward significance (order-level, p ≤ 0.10). No significant differences in abundance were observed for any other taxa at the phylum-, order-, and genus-level between the CD study – ileum and the non-CD control – ileum.

In colonic samples from CD patients, *Firmicutes* (phylum-level) were significantly less abundant in CD study – colon vs. the non-CD control – colon (MRA 39.5 (CI: 26.7.-52.3%) vs. 60.5% (CI: 48.1-72.8%), p=0.04). *Enterobacterales* (order-level) showed a significantly higher MRA of 24.6% (CI: 7.8-41.4%) in CD study – colon vs. 9.3% (CI: 2.4-16.3%) in the non-CD control – colon (p=0.03). *Bacteroides* and *Escherichia/Shigella/Pseudescherichia* (genus-level) were more abundant in CD study – colon vs. the non-CD control – colon (MRA 23.6% (CI: 14.4-32.1) and 22.7% (CI: 6.7-38.8) vs. 7.2% (CI: 2.8-11.6%) and 7.4% (CI: 1.0-13.8), p ≤ 0.05). *Tissierellales* (order-level) and *Prevotella* (genus-level) were found only in non-CD control – colon (0.0% (CI: 0.0-0.0) and 0.0% (CI: 0.0-0.0) vs. 4.3% (CI: -0.7-9.3) and 7.8% (CI: -2.2-17.8%), p ≤ 0.01). *Proteobacteria* (phylum-level) were more abundant in CD study – colon with a trend toward significance (MRA 29.4% (CI: 13.4-45.3%) vs. 14.8% (CI: 7.2-22.4%), p=0.08).

### 3.2 Microbial analysis of ileal samples from the CD non-affected control

**Figure 6:**
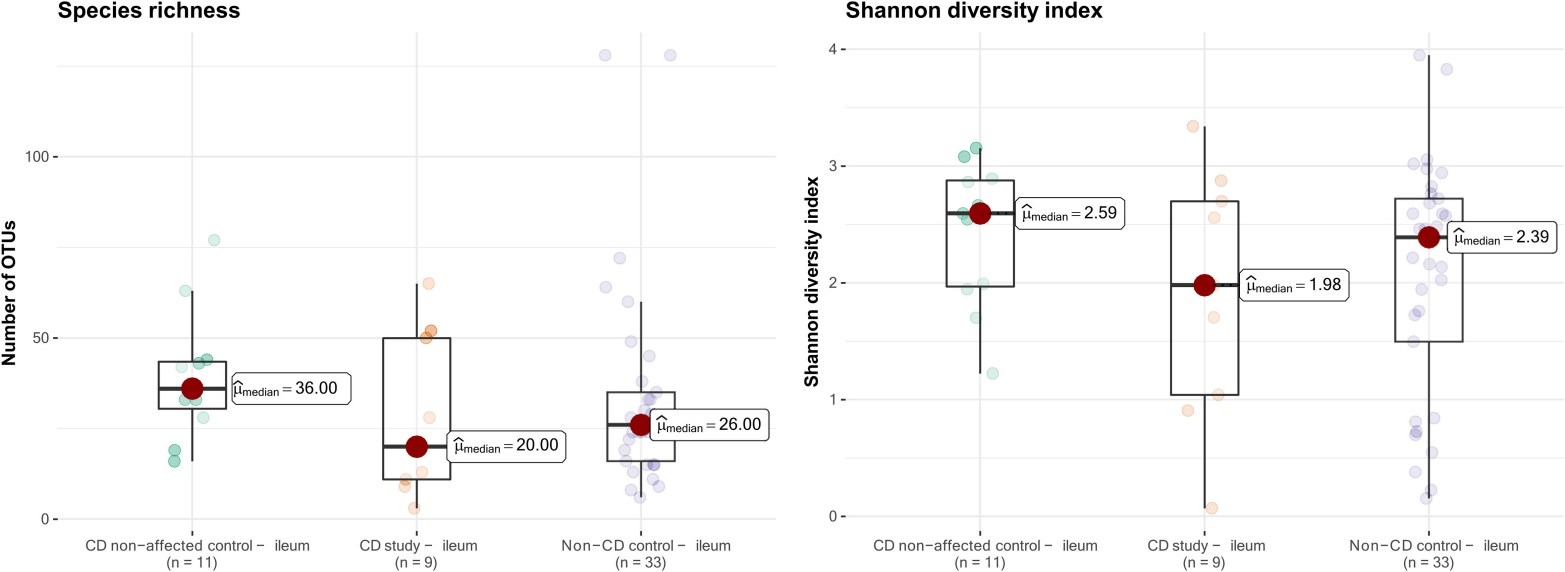
Species richness (left) and Shannon diversity index (right) of ileal samples from the CD non-affected control, CD study, and non-CD control. OTUs, operational taxonomic units.

The microbial composition of the second control group, CD non-affected control – ileum, was then analyzed. In this group, an overall median of 36.0 OTUs was found, and the median Shannon diversity index was 2.59 (**Figure 6**). No significant difference was observed in α-diversity for species richness and Shannon diversity index between CD, non-affected – ileum, and CD study – ileum or non-CD ileum (p>0.05).

In **Table 2**, the MRAs of the most abundant taxa from the control CD non-affected control – ileum are presented and compared to CD study – ileum and non-CD – ileum. Previously, significantly different abundances of taxa between CD study – ileum and non-CD control – ileum were only seen for *Tissierellales* (order-level) and for *Klebsiella* (genus-level, p ≤ 0.05, see 3.1.2). CD non-affected control – ileum, however, presents an individual microbial composition showing more differentially abundant taxa compared to each of these two groups.

**Table 2:** Mean relative abundances (MRAs) for the CD non-affected – ileum, CD study – ileum, and non-CD control – ileum groups.

	CD non-affected control - ileum	*p* (CD non-affected control - ileum vs. CD – ileum)	*p* (CD, non-affected – ileum vs. non-CD control – ileum)	CD study - ileum	Non-CD control - ileum	*p* (CD – ileum vs. non-CD control – ileum)
Phyla						
*Firmicutes*	46.8% (CI: 31.8-61.7)	0.33	0.30	34.5% (CI: 17.9-51.3)	36.4% (CI: 26.7-46.2)	0.70
*Proteobacteria*	28.9% (CI: 10.2-47.6)	*0.10	0.16	50.2% (CI: 25.6-74.9)	46.3% (CI: 34.1-58.4)	0.72
*Bacteroidetes*	11.0% (CI: -0.5-22.5)	0.82	0.73	10.7% (CI: 0.9-20.5)	9.3% (CI: 4.6-14.0%)	0.63
*Actinobacteria*	8.2% (CI: 3.4-13.0)	0.07*	0.02**	3.7% (CI: 0.6-6.9)	3.9% (CI: 2.4-5.4)	0.88
*Fusobacteria*	4.6% (CI: -0.1-9.4)	0.41	0.35	0.7% (CI: -0.1-1.6)	2.7% (CI: 0.4-5.0)	0.90
*Synergistestes*	0.1% (CI: -0.1-0.2)	0.33	0.54	0.0% (CI: 0.0-0.0)	1.2% (CI: -0.5-2.9)	0.59
Orders						
*Enterobacterales*	9.5% (CI: -2.8-21.7)	<0.01**	0.02*	39.9% (CI: 13.3-66.6)	32.7% (CI: 20.2-45.2)	0.30
*Lactobacillales*	20.4% (CI: 3.6-37.3)	0.71	0.12	16.7% (CI: 4.0-29.4)	11.3% (CI: 5.4-17.3)	0.35
*Bacteroidales*	10.7% (CI: -0.9-22.2)	0.55	1.00	10.7% (CI: 0.9-20.5)	9.0% (CI: 4.3-13.8)	0.49
*Clostridiales*	12.5% (CI: 4.2-20.7)	0.50	0.39	14.6% (CI: -0.2-29.5)	11.5% (CI: 5.9-17.2)	0.55
*Other*	8.9% (CI: 5.6-12.2)	<0.01**	0.03**	2.7% (CI: 0.7-4.9)	5.4% (CI: 3.1-7.6)	0.47
*Burkholderiales*	2.8% (CI: 0.2-5.5)	0.88	0.96	1.7% (CI: 0.8-2.7)	7.0% (CI: 1.3-12.6)	0.53
*Veillonellales*	2.3% (CI: -0.3-5.0)	0.88	0.52	2.3% (CI: 0.5-4.1)	5.4% (CI: 1.9-8.9)	0.55
*Pasteruellales*	7.4% (CI: -3.1-18.0)	0.60	0.23	3.7% (CI: -1.9-9.3)	2.8% (CI: -2.5-8.1)	0.72
*Tissierellales*	5.3% (CI: 1.8-8.9)	<0.01**	0.08*	0.0% (CI: -0.1-0.1)	3.9% (CI: 0.7-7.1)	0.01**
*Bacillales*	5.5% (CI: 3.9-7.1)	<0.01**	<0.01**	0.8% (CI: -0.4-1.9)	2.5% (CI: 1.1-3.9%)	0.08*
*Fusobacteriales*	4.6% (CI: 0.1-9.4)	0.41	0.35	0.7% (CI:-0.1-1.6)	2.7% (CI: 0.4-5.0)	0.90
*Pseudomonadales*	1.4% (CI: -0.7-3.5)	0.66	0.31	4.9% (CI: -6.3-16.1)	1.8% (CI: 0.2-3.4)	0.09**
*Actinomycetales*	1.7% (CI: 1-1-4.5)	0.15	0.32	1.1% (CI:-1.3-3.5)	1.4% (CI: 0.4-2.5)	0.58
*Rhizobiales*	6.5% (CI: -7.8-20.9)	0.50	0.31	0.0% (CI: 0.0-0.0)	0.0% (CI: 0.0-0.1)	0.86
*Rhodobacterales*	0.1% (CI: -0.1-0.4)	0.77	1.00	0.0% (CI: 0.0-0.0)	1.1% (CI: -1.1-3.2)	0.70
*Synergistales*	0.9% (CI: -0.1-0.2)	0.33	0.54	0.0% (CI: 0.0-0.0)	1.2% (CI: -0.5-2.9)	0.59
Genera						
*Esch./Shig./Pse.*	1.5% (CI: -0.8-3.9)	0.01**	0.03**	32.8% (CI:4.1-61.5)	23.9% (CI: 12:3-35.5)	0.36
*Bacteroides*	4.9% (CI: -0.7-10.5)	0.26	0.63	9.6% (CI: 0.1-19.0)	5.8% (CI: 2.4-9.2)	0.33
*Streptococcus*	16.5% (CI: 0.0-32.9)	0.13	0.04**	8.2% (CI: -3.0-19.4)	6.4% (CI: 1.7-11.1)	0.93
*Haemophilus*	6.9% (CI: -3.7-17.5)	0.60	0.36	3.7% (CI: -1.9-9.3)	2.8% (CI: -2.5-8.1)	0.72
*Clostridium*	5.6% (CI: -1.4-12.7)	0.60	0.18	2.9% (CI: -0.9-6.7)	1.6% (CI: 0.1-3.2)	0.74
*Klebsiella*	4.3% (CI: -1.4-10.1)	0.02**	0.39	0.0% (CI: 0.0-0.0)	5.8% (CI: -1.0-12.6)	0.04**
*Methylobacterium*	6.4% (CI: -7.9-20.8)	1.0	0.78	0.0% (CI: 0.0-0.0)	0.0% (CI: 0.0-0.0)	0.74
*Porphyromonas*	5.0% (CI: -2.4-12.4)	0.33	0.56	0.0 (CI: 0.0-0.0)	0.1% (CI: 0.0-0.3)	0.41

CD, Crohn’s disease; CI, confidence interval; Esch./Shig./Pse., Escherichia/Shigella/Pseudescherichia.

For genera, MRAs are presented in cases where the value was ≥5.0% in at least one of the groups. P-values of the pairwise comparison of differences between the respective groups from the Wilcoxon rank-sum test are presented (**p-values ≤ 0.05; *p-values ≤ 0.10).

In both CD study – ileum and non-CD control – ileum, *Enterobacterales* (order-level) showed MRAs of > 30.0%, and no difference was seen between these two groups (p=0.30). In the CD non-affected control – ileum, *Enterobacterales* were distinctly less abundant compared to these groups with an MRA of 9.5% (CI: -2.8-21.7, p ≤ 0.05). *Bacillales* (order-level) were most abundant in the CD non-affected control – ileum (MRA 5.5% (CI: 3.9-7.1), p<0.01) and *Escherichia/Shigella/Pseudescherichia* (genus-level) were distinctly less abundant (1.5% (CI: -0.8-3.9, p ≤ 0.05) compared to the other groups.

Also in this analysis, compared to the CD study – ileum, *Tissierellales* (order-level) and *Klebsiella* (genus-level) were more abundant in the CD non-affected control – ileum (MRA 5.3% (CI: 1.8-8.9) and 4.3% (CI: -1.4-10.1) vs. 0.0 (CI:-0.1-0.1) and 0.0% (CI: 0.0-0.0), p=0.02).

Compared to the non-CD control – ileum, *Actinobacteria* (phylum-level) and *Streptococcus* (genus-level) were more abundant in the CD non-affected control – ileum (MRA 8.2% (CI: 3.4-13.0) and 16.5% (CI: 0.0-32.9) vs. 3.9% (CI: 2.4-5.4) and 6.4% (CI: 1.7-11.1), p ≤ 0.05).

## 4 Discussion

The importance of understanding microbial changes in CD patients has been acknowledged, and several studies to further comprehend these changes have been conducted. However, CD is a chronic, heterogeneous disease with multiple subsets of patients in terms of duration, affected regions, and severity of the disease, as well as different treatment regimens. In this context, the homogeneity of the investigated groups of patients poses a substantial challenge. Additionally, methodological heterogeneity in terms of fecal or mucosal tissue sampling limits the comparability of results (Wright et al., 2015; Zhuang et al., 2021).

The study group of CD patients (CD study) in this study consists solely of patients who underwent surgery for ileocecal CD after a median time since diagnosis of 12 years and thus represents a defined subset of patients with severe disease (**Table 1**). The main analysis compares the microbial composition of this study group (CD study) with that of non-CD patients (non-CD control). For microbial analysis, tissue samples obtained during surgery were used in all cases, and samples from corresponding regions (terminal ileum and right-sided colon) were analyzed individually and compared between groups.

In our presented study, *Firmicutes* were significantly less (MRA 39.5 vs. 60.5%, p=0.04) and *Proteobacteria* were more abundant, with a trend toward significance in CD study – colon compared to the non-CD control – colon (MRA 29.4% vs. 14.8%, p=0.08) at the phylum-level. This microbial compositional change of a higher abundance of *Proteobacteria* at the expense of *Firmicutes* in CD patients compared to non-CD patients has been noted in the existing literature (Wright et al., 2015; Nishino et al., 2018; Vester-Andersen et al., 2019) An increase in *Proteobacteria* accompanied by a decrease in several members of *Firmicutes* has been associated with endoscopic recurrence of CD following surgery (Sokol et al., 2020) and lower rates of *Firmicutes* in CD patients have been noted in relapsers vs. non-relapsers after infliximab withdrawal (Rajca et al., 2014). It remains ambiguous whether these changes simply accompany active disease or represent a footprint of patients with severe disease course. Vester-Andersen et al. compared the taxonomic composition of fecal samples from CD patients after seven years of clinical follow-up (Vester-Andersen et al., 2019). In their study, the same taxonomic phylum-level shift was associated with an aggressive course of CD (≥3 courses of systemic steroids and/or biologic therapy and/or surgical resection during the previous 7 years of follow-up) compared to the non-aggressive course of CD (p ≤ 0.05). When comparing active with inactive CD, however, this shift was not observed in the study by Vester-Andersen et al. (2019). Nishino et al. found the same phylum-level change while stating that the patients included in their study mainly presented with mild disease activity (Nishino et al., 2018).

Within the elevated *Proteobacteria* phylum in CD study – colon, *Enterobacterales* were more abundant at the order level, and *Escherichia/Shigella/Pseudescherichia* were more abundant at the genus level (MRA 24.6% and 22.7% vs. 9.3% and 7.2%, p ≤ 0.05) compared to the non-CD control – colon. Consistently, an increase in *Escherichia* has been associated with CD at disease onset (Gevers et al., 2014), predominantly mild disease activity (Nishino et al., 2018), and areas of active inflammation in CD (Wright et al., 2015; Libertucci et al., 2018). *Bacteroides* (genus-level) were also more abundant in CD study – colon vs. non-CD control - colon (23.6% vs. 7.2%, p=0.01), which has been previously described in fecal samples during the active phase of CD compared to healthy individuals (Andoh et al., 2012). Therefore, these highlighted taxonomic shifts in CD found in our study are generally in concordance with the existing literature. However, their unequivocal attribution to a distinctly severe disease course, as represented by our study group (CD study), based on the existing literature, remains elusive.

Moreover, in our study, these highlighted considerable microbial shifts at the phylum-level, subsequently accompanied by distinct order- and genus-level changes, were only present in the comparison of colonic samples of the CD study vs. the non-CD control but not in ileal samples. In ileal samples, only slight changes in microbial composition were noted, represented by a lower abundance of *Tissierellales* (order-level) and *Klebsiella* (genus-level) in the CD study – ileum vs. non-CD control – ileum (MRA 0.0% and 0.0% vs. 3.9% and 5.8%, p ≤ 0.05). Our findings may suggest that in surgical CD patients at the time of surgery, the colon may be more susceptible to or associated with microbial alterations compared to the ileum when affected by CD. In contrast, several studies have demonstrated marked changes, including phylum-level shifts, of the mucosal ileal microbiome in surgical CD patients at the time of or after surgery compared to non-CD controls (Wright et al., 2017; Machiels et al., 2020). However, the different extent of change in the microbial composition depending on the affected location of CD in this study emphasizes the importance of specific regional analyses of microbial composition in order to fully comprehend microbial changes in CD. Methodologically, this aspect is not addressed by the analysis of fecal samples (Andoh et al., 2012; Rajca et al., 2014; Doherty et al., 2018; Vester-Andersen et al., 2019) or by the mucosal analysis of biopsies taken from the ileum only in ileocolic CD (Wright et al., 2017; Laffin et al., 2018; Machiels et al., 2020; Sokol et al., 2020). In the subsequent performed analysis, the second control group consisted of CD patients (CD non-affected control) with a median disease duration of 10 years (**Table 1**) who had previously received a protective ileostomy because of CD. Microbial analyses of these CD patients were performed on samples taken at the time of the closure of these ileostomies from the ileum unaffected by CD. In the main analysis, little microbial difference was present between the CD-affected ileum (CD study – ileum) and the non-CD control – ileum. In this subsequent analysis, however, the CD non-affected control – ileum showed several significant taxonomic differences at the phylum-, order-, and genus-level from both groups (CD study – ileum and non-CD control – ileum, see 3.2). These results underline that microbial changes in CD are not necessarily dependent on the current inflammation. Also, in previous literature, microbial differences have been noted between active vs. inactive disease CD (Clooney et al., 2021; Guo et al., 2022) and even in healthy co-twins of symptomatic IBD-twins (Brand et al., 2021).

Despite the encouraging results presented, our study has limitations. First, the cross-sectional study design did not allow longitudinal analyses with the possibility to more specifically analyze predictive patterns of the course of CD within the defined subgroup of included patients. Additionally, the group sizes investigated were limited, and our study group of CD patients (CD study) and non-CD controls were not age-matched. The study of a larger group of patients in combination with a longitudinal follow-up of the patient’s disease and microbial development would improve the power of the results and give more differentiated insights into the microbial changes during the course of CD. Nonetheless, this study presents the microbial analysis of the mucosa of affected and unaffected CD regions in a subset of patients homogeneous with respect to the severity of the previous disease course. The basis for these analyses was surgical tissue samples, which allowed a precise pairwise comparison of the respective regions with a control group of non-CD patients. Thus, the results contribute to the microbial composition of a well-defined subset of CD patients without confounding aspects of the fecal microbiome and regional microbial differences in the existing literature.

The remaining challenge for future studies is to disentangle which specific microbial changes generally accompany disease or even susceptibility, precede disease onset, are present in flares or long-standing diseases, and allow prediction of prospective disease severity (Wright et al., 2015; Ananthakrishnan, 2020; Aldars-Garcia et al., 2021; Zhuang et al., 2021; Guo et al., 2022). With regard to surgical CD patients, a deeper understanding of microbial changes may enhance the indication and the proper timing of surgery. Additionally, it may contribute to the decision on the necessary extent of surgical radicality, ranging from establishing diverting stomas through stricturoplasty to performing intestinal resections. Specific microbial profiles may even be used to identify diseases that are particularly prone to postoperative complications, such as anastomotic leakage, and thus be incorporated into surgical treatment decisions. Prospective, longitudinal studies that include sufficiently sized subsets of patient groups with different courses, severities, and treatment regimens of CD are warranted to further differentiate and comprehend microbial changes during the course of the disease.

## Data availability statement

The datasets presented in this study can be found in online repositories. The names of the repository/repositories and accession number(s) can be found below: NCBI repository (ID: 1048714; https://www.ncbi.nlm.nih.gov/bioproject/1048714), PRJNA1048714.

## Ethics statement

This study involving humans were approved by Ethics committee of the Charité—Universitätsmedizin Berlin, corporate member of Freie Universität Berlin and Humboldt-Universität zu Berlin (#EA4/165/18). This study were conducted in accordance with the local legislation and institutional requirements. The participants provided their written informed consent to participate in this study.

## Author contributions

FL: Conceptualization, Data curation, Formal analysis, Investigation, Methodology, Visualization, Writing – original draft. CK: Conceptualization, Data curation, Formal analysis, Investigation, Methodology, Project administration, Writing – review & editing. PM: Conceptualization, Data curation, Formal analysis, Investigation, Methodology, Visualization, Writing – original draft. RS: Conceptualization, Data curation, Formal analysis, Investigation, Methodology, Writing – review & editing. KB: Supervision, Writing – review & editing. CS: Conceptualization, Data curation, Formal analysis, Investigation, Methodology, Project administration, Supervision, Writing – original draft.
