# Supplementary figures and images for: Mucosal microbiome of surgically treated terminal ileal Crohn’s disease
Source: Front Cell Infect Microbiol. 2024 Jan 12;13:1324668. doi: 10.3389/fcimb.2023.1324668 (PMC10811112; doi:10.3389/fcimb.2023.1324668)

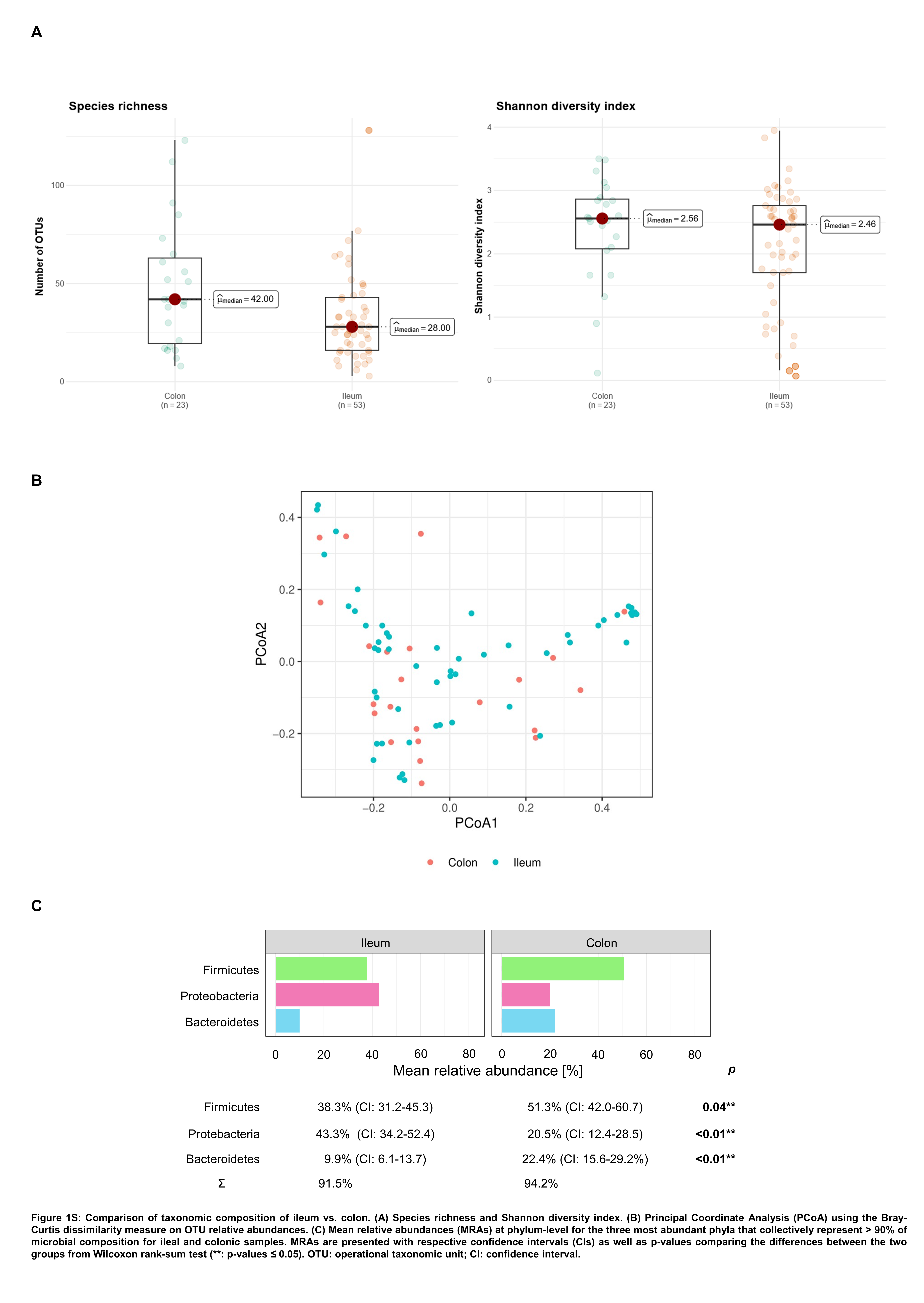

Supplement: Supplementary file 1 [file Image_1.tif]
